# Detection of dicistroviruses RNA in blood of febrile Tanzanian children

**DOI:** 10.1080/22221751.2019.1603791

**Published:** 2019-04-19

**Authors:** Samuel Cordey, Florian Laubscher, Mary-Anne Hartley, Thomas Junier, Francisco J. Pérez-Rodriguez, Kristina Keitel, Gael Vieille, Josephine Samaka, Tarsis Mlaganile, Frank Kagoro, Noémie Boillat-Blanco, Zainab Mbarack, Mylène Docquier, Francisco Brito, Daniel Eibach, Jürgen May, Peter Sothmann, Cassandra Aldrich, John Lusingu, Caroline Tapparel, Valérie D’Acremont, Laurent Kaiser

**Affiliations:** a Division of Infectious Diseases and Laboratory of Virology, University of Geneva HospitalsGeneva, Switzerland; b University of Geneva Medical SchoolGeneva, Switzerland; c Department of Ambulatory Care and Community Medicine, Lausanne University HospitalLausanne, Switzerland; d Swiss Institute of BioinformaticsGeneva, Switzerland; e Global Health Institute, School of Life Sciences, École Polytechnique Fédérale de LausanneLausanne, Switzerland; f Swiss Tropical and Public Health Institute, University of BaselBasel, Switzerland; g Ifakara Health Institute, Dar es Salaam, Tanzania; h Amana Hospital, Dar es Salaam, Tanzania; i Infectious Diseases Service, Lausanne University HospitalLausanne, Switzerland; j Mwananyamala Hospital, Dar es Salaam, Tanzania; k iGE3 Genomics Platform, University of GenevaGeneva, Switzerland; l Department of Genetic Medicine and Development, Faculty of Medicine of GenevaGeneva, Switzerland; m Department of Infectious Disease Epidemiology, Bernhard Nocht Institute for Tropical MedicineHamburg, Germany; n German Centre for Infection Research (DZIF), Hamburg, Germany; o Division of Tropical Medicine, 1st Department of Medicine, University Medical Center Hamburg-EppendorfHamburg, Germany; p Division of Infectious Diseases and Tropical Medicine, Medical Center of the University of Munich (LMU)Munich, Germany; q National Institute for Medical Research, Tanga Research Centre, Tanga, Tanzania; r Geneva Centre for Emerging Viral DiseasesGeneva, Switzerland

**Keywords:** Dicistrovirus, de novo analysis, sera, viremia, Tanzanian children

## Abstract

Fever is the leading cause of paediatric outpatient consultations in Sub-Saharan Africa. Although most are suspected to be of viral origin, a putative causative pathogen is not identified in over a quarter of these febrile episodes. Using a de novo assembly sequencing approach, we report the detection (15.4%) of dicistroviruses (DicV) RNA in sera collected from 692 febrile Tanzanian children. In contrast, DicV RNA was only detected in 1/77 (1.3%) plasma samples from febrile Tanzanian adults, suggesting that children could represent the primary susceptible population. Estimated viral load by specific quantitative real-time RT–PCR assay ranged from < 1.32E3 to 1.44E7 viral RNA copies/mL serum. Three DicV full-length genomes were obtained, and a phylogenetic analyse on the capsid region showed the presence of two clusters representing tentative novel genus. Although DicV-positive cases were detected throughout the year, a significantly higher positivity rate was observed during the rainy season.

This study reveals that novel DicV RNA is frequently detected in the blood of Tanzanian children, paving the way for further investigations to determine if DicV possibly represent a new agent in humans.

## Introduction

Infectious febrile illnesses are leading causes of paediatric outpatient consultations in Sub-Saharan Africa. For a large proportion of these cases, the microbial aetiology remains unknown even after extensive diagnostic panels have been applied; thus, it is suspected that new or divergent viruses that are not detectable by standard targeted diagnostics assay may be involved. High-throughput sequencing (HTS) coupled with de novo assembly offers the possibility of recognizing viral RNA or DNA in an unbiased fashion. HTS-based screenings in both vertebrate and non-vertebrate biological specimens [1–3] have shown that the diversity of viruses circulating in the animal world is larger than anticipated (including potential human infections such as astroviruses, reoviruses, pseudorabies viruses, and bornaviruses [4–9]), and that many viral families are shared by the two kingdoms, raising the possibility of yet to be discovered viral infections in humans [10].

Using HTS and a de novo assembly approach, we screened sera collected from febrile Tanzanian children for the presence of unrecognized viral agents. These children were enrolled in a large cohort investigating the clinical impact of point-of-care patient management system featuring an electronic algorithm based on host biomarkers [11]. After identifying an index case harbouring a full-length sequence of dicistrovirus (DicV), we used the complete original sequence obtained to extend screening across the 692 paediatric patients enrolled in this cohort, as well as 77 febrile adults included in a similar study [12]. *Dicistroviridae* are non-enveloped, positive-sense single-stranded RNA viruses belonging to the picorna-like viruses. DicV infect a wide spectrum of arthropods including species that are known to feed on human blood [13].

This report describes the detection of DicV RNA, including full-length genomes, in sera from Tanzanian children with acute febrile illnesses. The presence of DicV RNA was further confirmed by specific quantitative real-time RT–PCR assays as well as conventional sequencing. We also provide a description of epidemiological, demographic and clinical features associated with the presence of DicV sequences and propose strategies for further investigations to explore the role of this virus.

## Materials and Methods

### Study design

**Figure 1.**
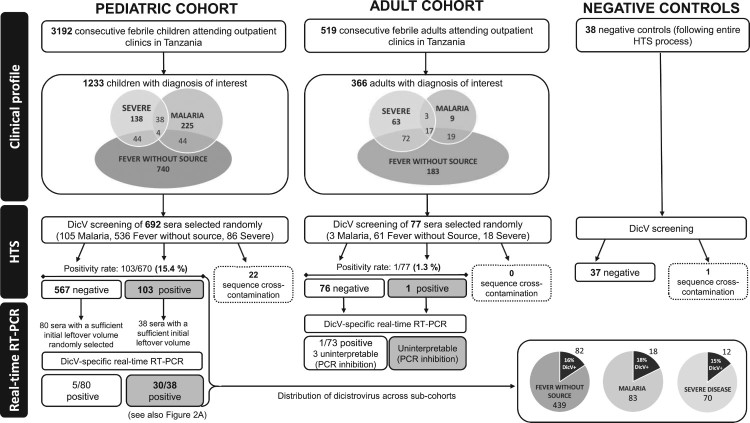
Study flow chart. Severe febrile illness is defined as having severe tachypnea (respiratory rate ≥97th percentile for age and temperature), severe tachycardia (heart rate ≥90th percentile for age and temperature), Hypoxemia (SaO2 < 90%), inability to tolerate oral fluids and/or neurological danger signs (such as coma or >2 convulsions in 24 h). The diagnosis of “fever without source” is attributed when the probable cause of a febrile illness cannot be determined by history or physical examination. The diagnosis of “malaria” is attributed in case of positive malaria-specific quantitative PCR, targeting multiple genomic copies of the conserved C-terminus of the var gene family (detection level: 0.1 parasite/µl). HTS: high-throughput sequencing, DicV: dicistrovirus, RT-PCR: reverse transcription PCR.

A total of 692 serum samples were selected from a larger paediatric cohort of 3192 patients (2 months to 5 years of age). Participants were consecutively recruited between December 2014 to February 2016 across nine outpatient clinics in Dar es Salaam (Tanzania) according to the inclusion criteria of “acute febrile illness” [11]. The 692 serum samples were randomized from a sub-cohort of patients with the diagnoses of “fever without source”, “severe febrile illness” or “malaria” as detected by PCR (Figure 1). This sub-cohort represents a group of particular clinical interest for expanded diagnostic screening. As sample randomization in children was limited by sample volume availability, we analysed the selection for potential bias. Selected samples were not significantly different for all key demographic variables (such as age and sex) nor for geographic (district) or temporal (season) features. In addition, 77 plasma specimens were randomly selected from an adult cohort of 519 patients. Adult patients were consecutively recruited between July 2013 to May 2014 across outpatient clinics in Dar es Salaam according to the inclusion criteria of “acute febrile illness” [12]. Samples were then randomly selected from a sub-cohort of interest with the same 3 diagnoses as above (Figure 1).

### Ethical approval

This study has received local IRB approval in Tanzania by the Ifakara Health Institute and the National Institute for Medical research (paediatric cohort: IHI/IRB/EXT/16-2015 and NIMR/HQ/R.8a/Vol. IX/1789; adult cohort: IHI/IRB/No: 12–2013 and NIMR/HQ/R.8a/Vol. IX/1561). IRB approval was also granted for analyses undertaken in Switzerland by the Ethikkommission Nordwest- und Zentralschweiz (paediatric cohort: EKNZ UBE-15/03 and adult cohort: EK 1612/13).

### De novo analysis and sequence analysis

Unbiased HTS protocols was performed on the 692 sera and 77 plasma specimens from the paediatric and adult cohorts, respectively. Using a de novo assembly approach (see below), an index case harbouring a full-length sequence of DicV was detected. This complete DicV sequence was used to extend screening across all specimens analysed by HTS.

The sample preparation for HTS analysis (i.e. specimen centrifugation, DNAse treatment and RNA extraction by TRIzol) was performed as previously published [14]. RNA libraries (TruSeq total RNA preparation protocol (Illumina, San Diego, US)) were multiplexed by 4 and run on a HiSeq 2500 (Illumina, San Diego, CA, USA). Raw data were low-quality and adaptor-trimmed using Trimmomatic (V0.33), human- sequence subtracted (GRCh38, gencode.V23) using snap-aligner (v1.0beta.23) and then analysed using the de novo genome assembly IDBA-UD software (v.1.1.3) [15]. Reads from raw data were mapped to complete-coding genomes obtained in previous steps. Cross-lane contamination was checked using a crosstalk rate of 0.3% [16]. Cross-lane contamination was detected for 22 paediatric samples which were subsequently excluded. The remaining 670 samples from individual patients were retained for further epidemiologic, demographic and clinical investigations (Table 1). The median of total number of read pairs obtained were 45’386’721 and 46’177’840 for DicV- and DicV+ patients, respectively, with a minimum of 15’760’060 read pairs obtained for DicV+ patients.

**Table 1.** Comparative analysis between dicistrovirus-positive and negative febrile children.

* *p* < 0.05, CRP: C-reactive protein, PCT: procalcitonin, HIV: human immunodeficiency virus

^†^ 36 cases with an unknown district excluded

^‡^ data on this symptom was only collected from children old enough to express the symptom/respond to the question

^§^ data limited by sample availability (only collected when clinically appropriate or insufficient sample volume)

^#^ PCT is an inflammatory marker, values larger than 0.5ug/ml have been associated to a higher risk of bacterial infection

To check potential reagent contaminants, 38 negative controls (DNase-free, RNase-free H_2_O) submitted to the whole HTS process were tested by de novo analysis (Figure 1).

**Figure 2.**
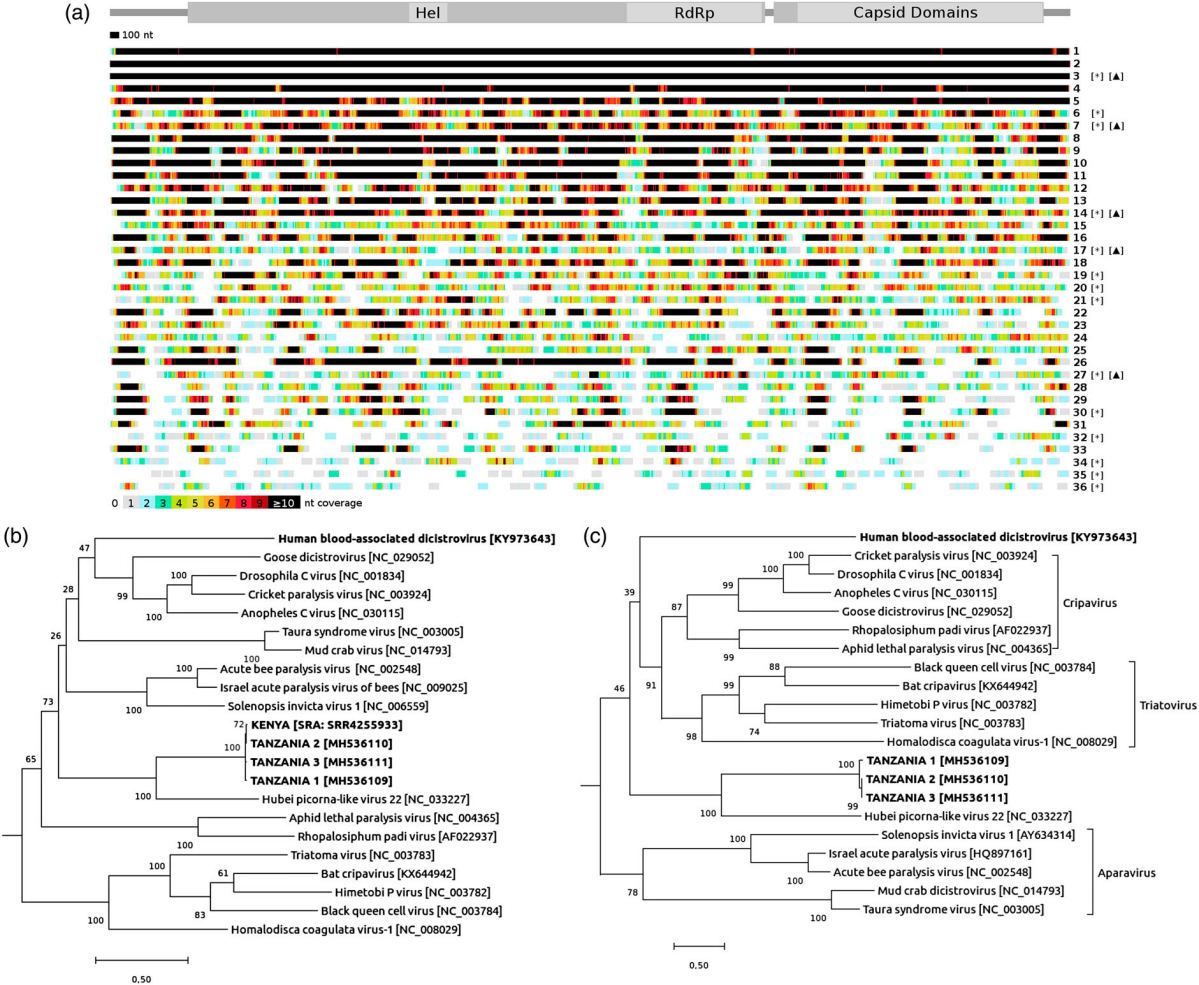
Analysis of Dicistrovirus sequences. (a) Location of DicV sequences with more than fifty percent DicV genome coverage obtained by de novo analysis for positive subjects. A schematic representation of the major ORFs of the DicV genome is featured at the top of the figure (Hel: helicase domain [pfam00910]; RdRp: RNA-dependent RNA polymerase domain [pfam00680]; Capsid domains include two Picornavirus capsid-like domains [cd00205], a DicV VP4 domain and CRPV capsid protein like domain [pfam08762]). On the right side is indicated when the presence of DicV could be verified by real-time RT-PCR (*) in sera with sufficient leftover volume and/or by RT-PCR followed by classical sequencing (▴). (b) Phylogenetic tree of the RdRp region of DicV. The 370-residue amino acid sequence considered for RdRp alignment is between position 1435 and 1804 of ORF1. Dicistrovirus sequences found in human blood are in bold font; the three complete coding sequences obtained in this study (TANZANIA 1–3), the Human blood-associated dicistrovirus sequence reported from a Peruvian patients with fever of unknown aetiology [21], and the KENYA sequence detected by our retrospective analysis in one pool (SRR4255933) from a study that investigated the plasma virome of febrile adult Kenyans [22]. (c) Phylogenetic tree of the capsid region of DicV. The 791-residue amino acid sequence considered for capsid alignment is between position 73 and the terminus of ORF2. Dicistrovirus sequences found in human blood are in bold font; the three complete coding sequences obtained in this study (TANZANIA 1–3), and the Human blood-associated dicistrovirus sequence reported from a Peruvian patients with fever of unknown aetiology [21].

After removal of PCR duplicates, only sequences with a coverage ≥ 5x for each individual nucleotide were considered for further analysis (Figure 2a-c). Three complete coding sequences were obtained and submitted to GenBank (accession no. MH536109-11). Phylogenetic trees were inferred for the RNA-dependent RNA polymerase (RdRp) (Figure 2b) and the capsid regions (Figure 2c) using a 500-replicate Maximum Likelihood method based on the LG model [17]. Analyses were performed with a Gamma distributed rate model with invariant sites using MEGA X software [18].

### Specific dicistrovirus real-Time RT–PCR

The aligned DicV sequences obtained from de novo analysis were used to design two specific real-time RT–PCR assays (Table 2): the quantitative DicV-Capsid and the semi-quantitative DicV-ORF1/RdRp. Samples in which DicV was identified by HTS analysis and for which sufficient volume remained (*n* = 38/103) were checked by these real-time RT–PCR assays. Briefly, the viral genomes were extracted using the NucliSENS easyMAG (bioMérieux, Geneva, Switzerland) nucleic acid kit from 190 ul of sera spiked with 10 ul of standardized canine distemper viruses, as previously described [19]. Nucleic acids were eluted in 25 ul and then screened for the presence of DicV using the DicV-Capsid real-time quantitative assay (upper and lower limits of quantification are 1.32E8 and 1.32E3 estimated viral RNA copies/mL, respectively) and the DicV-ORF1/RdRp assay. Both assays were performed using the one-step QuantiTect Probe RT–PCR Kit (Qiagen, Hombrechtikon, Switzerland) in a StepOne Plus instrument (Applied Biosystems, Rotkreuz, Switzerland) under the following cycling conditions: 50°C for 30 min, 95°C for 15 min, 45 cycles of 15 s at 94°C, and 1 min at 55°C.

**Table 2.** Primer and probe sequences. Fwd: primer forward, Rev: primer reverse, AT: PCR annealing temperature, NT: nucleotide.

	Assay	Sequence (5’ to 3’)		Final [uM] Fwd/Rev/Probe	
Real-time RT-PCR	DicV-Capsid	Fwd	GGGATGCAYGCTCTTGTAAATT	0.9/0.9/0.25	
		Rev	CRCACACCAACGAAACCA		
		Probe	FAM - ATGTCAGGCATTGAAC – MGBNFQ		
	DicV-ORF1/RdRp	Fwd	AGCTGGCGATTTCTCAAATTATG	0.9/0.9/0.25	
		Rev	TCGTCGTAAAATTCGTTGATTATGTC		
		Probe	FAM - TGGTACATTGCATCCCGAYATCTTATTGG -TAMRA		
** **	Target region	Sequence (5’ to 3’)		Productsize (nt)	AT (°C)
Classic RT-PCR	ORF1	Fwd1	TGAAGATTTCGAAGGAAAAATGG	427	50
		Rev1	ACGCGTTCTATTTATTCCTCGA		
		Fwd2	ATGATTCCAATACCGGATGTTGAGA	380	50
		Rev2				ATTCCTCGAATAAATTGTGAACACG

### Additional Tanzanian cohort screened for DicV by real-Time RT–PCR

The study was designed to assess the role of rickettsial infection as a source of fever amongst paediatric and adult outpatients in North-Eastern Tanzania. The study was based at the National Institute for Medical Research’s (NIMR) field station in Korogwe, Tanga Region. The field station is associated with Korogwe District Hospital (KDH).

Between 1 December 2017 and 30 April 2018, patients of five years and above presenting to the Outpatient Department of KDH with fever (>37.5°C tympanic) or a reported history of fever within the last three days were recruited. Blood was taken on day 0 and day 28 to establish a serological diagnosis of acute rickettsial infection. Amongst 336 patients recruited for the study, 271 (81%) completed the follow up on day 28. Amongst those patients with paired sera available (at least 500 μl per serum), 100 individuals (range from 6 to 80 years of age, median age = 27 years) were randomly selected for DicV screening by the DicV-Capsid and the DicV-ORF1/RdRp real-time RT–PCR assays. Among the 100 screened patients, 60 were > 16 years of age (median age = 45 years of age) and 40 were between 6 and ≤ 16 years of age (median age = 10 years of age). The study was approved by the National Ethics Review Committee of the National Institute for Medical Research, Tanzania.

### Respiratory and dicistrovirus detection in respiratory specimens

Of the 21 paediatric patients with respiratory symptoms (Table 1) a nasopharyngeal swab specimen (NPS) was collected for 16 subjects (3 DicV+ and 13 DicV- by HTS). The 16 NPS were extracted using the NucliSENS easyMAG (bioMérieux) and screened for the presence of DicV (using the DicV-Capsid and the DicV-ORF1/RdRp real-time RT–PCR assays) and a large panel of respiratory viruses using the FTD® Respiratory pathogens 21 commercial kit (Fast Track Diagnostics, Sliema, Malta).

### Statistical analysis

Statistical analysis compared various demographic (age, sex), epidemiological (seasonality, location) and clinical (signs, symptoms, co-morbidities and outcomes) parameters between the HTS DicV- (*n* = 567) and DicV+ (*n* = 103) paediatric sera samples. Analyses were undertaken using Stata (StataCorp. 2015, College Station, TX, USA) and associations between the variables of interest and the presence of DicV sequences were assessed using chi-squared, Spearman correlation and Kruskel-Wallis tests, as appropriate. Results are presented as means with 95% confidence intervals, frequencies and percentages. A two-sided *P*-value of <0.05 was considered statistically significant.

## Results

### Dicistrovirus identification and screening

Of the 670 sera (from 670 individual paediatric subjects), DicV genome sequences were detected in 103 samples (15.4%) (Figure 1; total number of DicV mapping reads ranging from 2 to 121’610, mean = 3’379, median = 240). To confirm the HTS analysis findings, all positive sera with sufficient leftover volume (*n* = 38) were assessed with the quantitative DicV-Capsid and the DicV-ORF1/RdRp real-time RT–PCR assays. Thirty/38 (79%) sera were confirmed DicV+ by real-time RT–PCR (21/30 by the two assays, 9/30 by one assay). The corresponding numbers of DicV-specific reads ranged from 6 to 121’610 for real-time RT–PCR positive sera. Estimated viral load ranged from < 1.32E3 up to 1.44E7 viral RNA copies/mL of sera (median viral load = 5.67E3 viral RNA copies/mL). The 8 sera not confirmed by real-time RT-PCRs had ≤ 280 DicV-specific reads (median = 16 reads) obtained by the HTS analysis. Of these 8 sera, 7 had a sufficient initial volume for further testing and prepared in two dilutions (1:2 and 1:10) to remove potential PCR inhibitors. None was found positive by real-time RT–PCR. In parallel, 80 sera found DicV-negative by HTS analysis were randomly selected throughout the entire recruitment period of 2015 and assessed by the DicV-Capsid and DicV-ORF1/RdRp real-time RT–PCR assays; 5 were found positive (4 sera collected in February and 1 in July; viral load ranged from < 1.32E3 to 2.11E4 viral RNA copies/mL of sera) suggesting that the DicV prevalence of 15.4% observed by HTS represents a minimal estimate.

In contrast to the HTS analysis results obtained with the paediatric cohort, the presence of DicV RNA was detected in only one of 77 (1.3%) plasma specimens from the adult cohort (2 reads, real-time RT-PCRs were performed but the results were not conclusive due to the presence of a very strong PCR inhibition) (Figure 1). In parallel, the 76 plasma found DicV-negative by HTS analysis were assessed by the DicV-Capsid and DicV-ORF1/RdRp real-time RT–PCR assays; 1 was found positive (collected in November 2013; viral load was < 1.32E3 viral RNA copies/mL of plasma). Of note, among the 38 negative controls submitted to the whole HTS process, none was found positive for DicV.

### Dicistrovirus sequence analysis

The phylogenetic trees based on the RdRp and the capsid regions (Figure 2b,c) show that the Tanzanian DicV sequences share the closest amino acid identities with the Hubei picorna-like virus 22 (NC_033227.1) for RdRp (53% identity) and capsid (32% identity) proteins. Based on the divergence of sequences in the capsid region, the three Tanzania sequences (MH536109-11) indicated the presence of two clusters representing tentative novel genus (Figure 2c). Indeed, in the capsid region, an amino acid identity of 95.2% is observed between the two clusters, while an amino acid identity of 99.46% is present in the RdRp region.

### Demographic and epidemiological analysis

A comparative analysis of demographic and clinical features between the DicV+ and DicV- paediatric populations are listed in Table 1. While there was a similar sex ratio between DicV+ and DicV- groups (61% vs 55%, *p* = 0.25), DicV+ patients had a lower mean age compared to the DicV- group (16.4 vs 18.9 months, *p* = 0.02).

**Figure 3.**
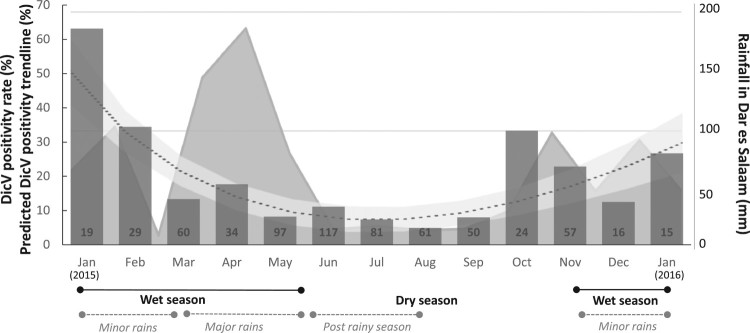
Monthly Dicistrovirus-positivity rate in febrile children. Bar graphs represent the positivity rates (%) (Left Y axis) of DicV RNA across each month of the study. Too few specimens (*n* = 10) were collected in December 2014 and February 2016 to provide reliable positivity rates, and, thus, are not represented on the graph. The total number of sera collected appear in the base of each bar. A dotted line with shaded CI95% shows a fractional polynomial trendline for predicted DicV positivity (also featured on the left Y axis). The rainfall recorded in Dar es Salaam (mm; adapted from https://www.worldweatheronline.com/dar-es-salaam-weatheraverages/dar-es-salaam/tz.aspx) over the patient recruitment period appears as a shaded graphic in the background (Right Y axis). Corresponding seasons are indicated below.

A monthly distribution analysis shows significantly higher prevalence of positive cases during the wet season (minor rain period) than during the dry season (*p* < 0.001, Table 1). Indeed, 45.6% of DicV infections occurred in the minor rain period (47/103, Figure 3). Significant geographic clustering occurred across three districts in Dar es Salaam (*p* > 0.001). Whilst the Kinondoni and Temeke districts had a 12 and 13% DicV prevalence, respectively (51/417 and 20/151, respectively), infections were 3-fold more frequent in the Ilala district (39%, 26/66).

### Clinical analysis

Overall, DicV presence did not vary significantly among the 3 major sub-cohorts of fever without source (16%), malaria (18%) and severe febrile illness (15%) (Figure 1).

Clinical features such as axillary temperature, cough, respiratory distress, vomiting, diarrhoea, dysuria, any ear or eye problems, dermatological symptoms, and clinical danger signs were not significantly different between DicV+ and DicV- paediatric patients (Table 1). The presence of DicV was not associated with other concomitant infections such as malaria, HIV or urinary tract infections, nor was it significantly different in patients with common co-morbidities such as malnutrition, anaemia or sickle cell disease. Outcomes and severity measures were also not affected. While the inflammatory marker C-reactive protein (CRP) was not significantly different among the groups, procalcitonin (PCT) was significantly higher in the presence of DicV (9ug/ml vs 4.9ug/ml, *p* = 0.04), where a level of >0.5ug/L was 18% more frequent in DicV+ patients (*p* = 0.01).

**Figure 4:**
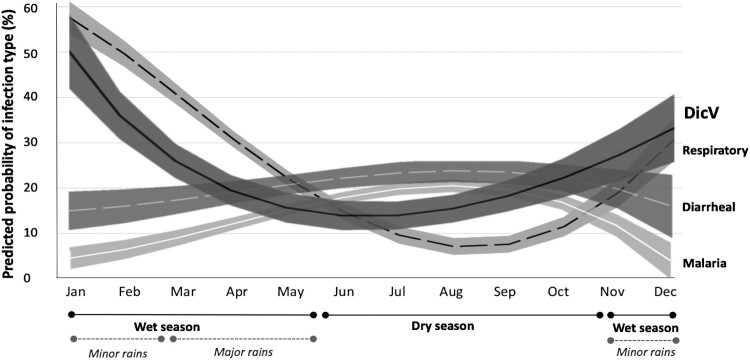
Temporal distribution of diseases representing the major modes of infectious transmission (airborne, fecal-oral and mosquito-borne) compared to DicV prevalence. The mean predicted prevalences (fractional polynomial trendlines) and their 95% confidence intervals (shaded areas around trendlines) are depicted for the probability of having the diagnoses of 1: “Respiratory infection” (dashed black line), 2: “Diarrheal disease” (white dashed line) and 3: Malaria (white solid line). The predicted prevalence of DicV is shown as a solid black line. The months of the entire 2015 recruitment period are depicted. Seasons are indicated below. DicV: Dicistrovirus.

The temporal distribution of diseases representing the major modes of infectious transmission was compared to DicV prevalence (Figure 4). The analysis revealed a temporal symmetry between the predicted probabilities of DicV infection and respiratory disease, which both peaked during the wet season. This is in contrast to both mosquito-vectored disease (malaria) and diseases usually transmitted via the fecal-oral route (diarrheal diseases) which peak in the post-flood and dry season. A total of 21 paediatric patients presented with respiratory distress (Table 1). For 16 subjects (13 DicV- and 3 DicV+ by HTS) a nasopharyngeal swab (NPS) sample was collected and screened by real-time (RT-)PCR for DicV and a large panel of respiratory viruses. Of the 13 DicV- patients, 11 were found positive for a respiratory virus in their respective NPS samples (three human parainfluenza virus type 3, three human rhinovirus, two human parainfluenza virus type 2, two human coronavirus HKU1 and one human parechovirus; no viral co-infections were observed). Of the three DicV+ patients, one respiratory virus was detected (human rhinovirus). Regarding DicV screening, none of the 13 DicV- patients had DicV sequences detectable in their NPS samples. In contrast, one NPS of the three available from the DicV+ patients was also found positive for DicV sequences (low viral load, no viral co-infection observed). This patient had DicV sequences detectable in serum by HTS which was confirmed by specific real-time RT–PCR assay (with an estimated viral load of 1.2E5 viral RNA copies/mL of serum).

## Discussion

Using an unbiased HTS approach and de novo assembly analysis, we identified distinct novel DicV sequences in 15.4% of paediatric patients attending an outpatient clinics for a febrile illness. Most DicV+ sera contained a large number of reads and a significant proportion of the DicV genomes was thus frequently obtained (Figure 2a), including full-length genomes in 3 cases. These HTS-based findings were subsequently verified by two specific in-house real-time RT–PCR assays in the 38 samples which had sufficient leftover sera (Figure 1) and by conventional sequencing in 5 samples selected for their high viral load (100% nucleotide identity, data not shown). Positivity in 30 of 38 (79%) cases could be confirmed by real-time RT–PCR with a median of 5.7E3 viral RNA copies/mL of sera and, in some cases, reached up to 1xE7 viral RNA copies/mL, supporting a significant viral load. In contrast to children, only one of 77 adults was found to be DicV-positive by HTS from a parallel cohort, suggesting that children could represent the predominant susceptible population. However, this latter observation should be interpreted with caution since a potential temporal bias cannot be ruled out (sampling times for the paediatric and adult cohorts are not overlapping). In order to expand our screening we tested by real-time RT–PCR 100 paired sera from adult and paediatric patients enrolled in a North-Eastern Tanzania cohort (Tanga region) between December 2017 and end of April 2018; we did not identify any DicV positive cases. However, not only a restricted number of adult (*n* = 60) and paediatric (*n* = 40) cases could be tested in this later cohort but also the paediatric populations studied in the Tanga and Dar es Salaam cohorts are different (e.g. age of enrolment) and limit our conclusions.

We also observed a clear seasonal distribution with clustering in the minor rain period of the wet season while few cases observed during the dry season (Figure 3). Taken together, these observations point toward the potential identification of a previously uncharacterized viral agent in human blood. This represents only a first step since we could unfortunately not have access to follow-up samples or any other validation cohort. According to Lipkin proposal concerning the levels of certainty in pathogen discovery, our finding fulfil the Level 1 criteria [20].

Whatever the molecular technology used, the discovery of new viral genomic sequences in body fluids must first exclude the possibility of contamination and/or spurious bioinformatics results particularly when HTS is used. In the present study, contamination has been carefully considered and is considered unlikely for the following reasons: Firstly, none of the 38 HTS negative controls that followed the entire HTS procedure revealed any DicV sequences, excluding the possibility of reagent contamination. Secondly, DicV positivity by HTS analysis was successfully cross-validated by separate extraction and real-time RT–PCR assays in 30/38 DicV+ patient sera with sufficient leftover volume, excluding the probability of spurious bioinformatics results and further eliminating contamination of HTS reagents (especially considering the absence of any common reagent between the HTS and real-time RT–PCR protocols). Five sera with the highest number of DicV reads were confirmed by conventional RT–PCR sequencing (Figure 2a; Table 2). The viral loads detected by real-time RT–PCR corresponded in most cases to the HTS read coverage and ranged from < 1.32E3 up to 1xE7 viral RNA copies/mL; this viral load heterogeneity speaks against a common and systematic source of contamination and would rather be compatible with the expected kinetics of an acute viral infection. The sequence heterogeneity (amino acids identity is 97.8% between the 3 DicV full-length sequences MH536109-11) found across DicV+ samples indicate different origins and speaks against a unique viral RNA source or spurious bioinformatics findings. Additionally, a phylogenetic analyse on the capsid region show the presence of two DicV clusters (amino acid identity was 95.2%) without any evidence of co-detections. Although only observed in a single case in blood samples, the presence of the DicV genome was previously reported in a Peruvian cohort of patients with fever of unknown aetiology [21]. In comparison to the Tanzanian sequences, the Peruvian DicV viral sequence showed amino acid identities as low as 35.5% and 22% in the RdRp and the capsid regions, respectively, confirming that they belong to different genera (Figure 2b,c). Finally, when analysing publicly available sequence databases from a recent study that investigated the plasma virome of febrile adult Kenyans [22], and thus has absolutely no link with our investigations, we identified 2’290 DicV reads covering 96.1% (5’350 nt) of ORF1 and 97.5% (2’525 nt) of ORF2 in one pool (SRR4255933). For ORF1, the covered sequence showed 95.4–96.4% nucleotide identity and 98.9–99.2% amino acid identity compared to the Tanzanian DicV sequences. ORF2 had similar results, with 91.5–97.5% nucleotide identity and 94.5–99.2% amino acid identities. The phylogenetic tree of the RdRp region shows that the Kenyan sequence clusters with the Tanzanian ones (Figure 2b), which represents an additional argument for the presence of this DicV novel genus in this geographic region.

Of note, specimens with as few as 6 DicV-specific reads could be confirmed by real-time RT–PCR, whereas 8 with a limited number of reads (≤ 240, median = 16) could not be confirmed. This latter result could be explained either by a higher sensitivity of the HTS in specific situations (particularly in relation to the additional freeze–thaw cycle for the real-time RT–PCR) and/or by the presence of primer-probe mismatches (this hypothesis could not be checked since only a very limited number of reads were obtained). The number of reads obtained through HTS provides a rough estimate of the viral load and depends on various factors, such as cellularity, the presence of other microorganisms and the efficiency of the library preparation [23].

Since dicistroviruses have been observed in a large spectrum of arthropods worldwide, one hypothesis raised by our findings is whether DicV could represent a viral agent or reflects a viral RNA transmission through contact with vector that still has to be discovered. Indeed, the seasonality clustering observed in this study, may suggest vector-borne transmission although others transmission routes cannot be ruled out. A zoonotic reservoir is also possible since DicVs have been detected in stools from various non-human primates (such as gorillas [24]) as well as giant pandas [25], and bats [26], which also raises the possibility of a faecal-oral or sylvatic transmission cycle. Furthermore, the presence of DicV sequences were recently reported in the blood of a fruit bat captured in 2015 from the Republic of Congo [27]. In humans, unbiased sequencing analysis of stool samples collected from 35 South Asian children presenting with non-polio acute flaccid paralysis revealed the presence of DicV in 3 subjects coinfected with other viruses [28]. Another study reported DicV sequences in stool samples from Australian children with acute diarrhoea [29]. In both studies, whether the presence of DicV in these stools resulted from a viral replication in the human gastrointestinal tract or from environmental contamination was not determined. At the in vitro level, the cellular tropism of DicV has been investigated using the Taura syndrome virus (TSV): a DicV that infects shrimp. Exposing various primate cells to this virus revealed a range of susceptibilities, where RD, Hep-2C and BGM cells were considered vulnerable to infection [30], while FRhK-4, MA-104 and BGMK remained non-permissive [31]. Both animal models as well as reverse genetics approaches will be needed to further investigate the tropism and likely replication sites of DicV in humans.

The present cohort offered the unique opportunity to assess demographics, symptoms and signs associated with the presence of DicV RNA. At this time, comparative analysis between DicV-positive and negative patients (HTS analysis) did not reveal any significant clinical differences, although the positive patients were significantly younger. The presence of DicV was not associated with any other co-infections (such as malaria, HIV or skin infections) nor was it linked to any other comorbidities. However, our cohort specifically enrolled patients at the outpatient level who presented unspecific febrile illnesses and thus covered a homogenous spectrum of clinical syndromes from which to draw conclusions. In the future, larger and more diverse cohorts should be screened, including more severe hospitalized children and asymptomatic blood donors.

In contrast to the paediatric population, DicV RNA was infrequently detected in adults. Whether this finding reflects acquired protective immunity in adults, a more frequent transmission from the vector, a greater susceptibility in the paediatric population or as mentioned above a temporal bias needs to be evaluated. A recent study investigated the seroprevalence of antibodies against another DicV from another genus named Triatoma virus (TrV) [32]. TrV is known to infect the vector responsible for Chagas disease transmission. Their findings revealed a high prevalence of anti-TrV antibodies against the TrV structural proteins in human sera of subjects living in Argentina, Bolivia and Mexico and suggested a passive transmission of the virus (i.e. absence of viral replication in human hosts). To fully investigate the possible transmission of DicV, future serological investigations should be carried out on a more diverse collection of symptomatic and asymptomatic adult and paediatric cohorts from varied geographical regions inside. At this time, we may raise the hypothesis of a vector-borne transmission and that, among the potential vectors, triatomines should be considered.

While this study does not establish the pathogenicity or even a true human infection, our findings suggest that DicV could represent a novel agent to consider in humans. We lack an afebrile cohort to assess the association. Other than the small increase in the inflammatory marker, PCT, we could not identify any specific clinical features, diagnoses or co-morbidities associated to DicV infection despite the detailed clinical database gathered at the time of sample collection. Elevated PCT is commonly associated (although not strongly) with bacterial infections; it may be increased either directly by bacterial endotoxins and lipopolysaccharides or indirectly by inflammatory mediators (such as tumour necrosis factor-alpha, interleukin-6, interleukin-1). Mediators of viral infection (interferon-gamma) are thought to attenuate PCT levels. Thus, its increase in DicV+ patients could be an indication that DicV may have a pathological immunomodulatory effect on its human host or an as yet unidentified association to another bacterial infection. However, given the small size effect size, no clinically relevant conclusions could be drawn from this observation. Notably, the de novo analysis performed on all sera and plasma specimens (from the paediatric and adult cohorts respectively) revealed the presence RNA sequences from viruses known to infect humans (Supplementary Figure S1). Of the 103 paediatric patients who were DicV+ by HTS, only ten presented a co-detection with sequences from these known human RNA viruses (two human pegivirus-1, two enteroviruses, four rhinoviruses and two rotaviruses). Human pegivirus-1 sequences were also detected in the plasma of the unique DicV+ adult patient by HTS. Studies in larger and more clinically diverse patients (including asymptomatic subjects) are needed to clarify whether DicV is pathogenic or if it should rather be considered as a member of the commensal blood virome, similarly to torque tenovirus or human pegivirus. Screening other types of samples will also help to define the in vivo tropism of DicV.

In conclusion, our findings that DicV RNA is frequently detected in the blood of Tanzanian children deserve attention. This study provides the first step toward a possible new agent in human, although without proven viral replication and open the door to further investigation to confirm and contextualize these results by screening other populations, and fulfilling all or part of Koch’s postulates.

## Supplementary Material

Supplemental Material
