# Supplementary figures and images for: Detection of dicistroviruses RNA in blood of febrile Tanzanian children
Source: Emerg Microbes Infect. 2019 Apr 19;8(1):613–23. doi: 10.1080/22221751.2019.1603791 (PMC6493270; doi:10.1080/22221751.2019.1603791)

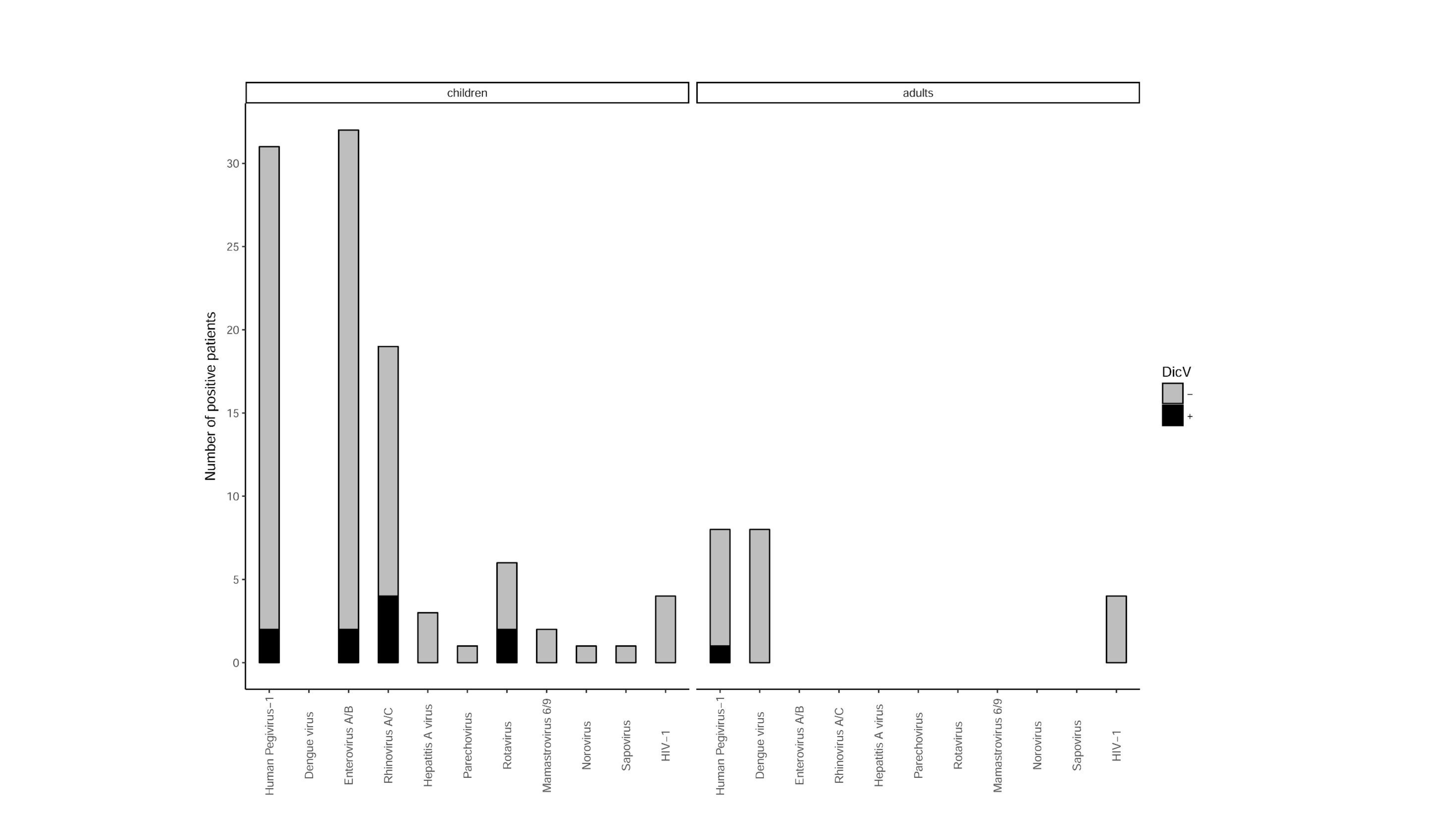

Supplement: Supplemental Material [file TEMI_A_1603791_SM7645.gif]
